# Facile and Sustainable Synthesis of Erythritol bis(carbonate), a Valuable Monomer for Non-Isocyanate Polyurethanes (NIPUs)

**DOI:** 10.1038/s41598-019-46314-5

**Published:** 2019-07-08

**Authors:** Patrick-Kurt Dannecker, Michael A. R. Meier

**Affiliations:** Laboratory of Applied Chemistry, Institute of Organic Chemistry (IOC), Karlsruhe Institute of Technology (KIT), Straße am Forum 7, 76131 Karlsruhe, Germany

**Keywords:** Sustainability, Organocatalysis

## Abstract

Recently, R. Mülhaupt *et al*. introduced the first high yielding synthesis of erythritol bis(carbonate) from erythritol with diphenyl carbonate (DPC) as reagent. They utilized it as monomer for the synthesis of non-isocyanate polyurethanes (NIPUs). Here, we present a significantly more sustainable procedure for the carbonate formation regarding solvent, carbonyl source, reaction temperature, reaction time, reduced pressure during the reaction, simplicity of the workup as well as recycling of reagents. Catalysed by triazabicyclodecene (TBD), dimethyl carbonate as solvent as well as reagent leads to selective product formation and facile product separation by filtration. After addition of new starting materials, the mixture of catalyst and DMC was reused up to 8 times without loss of catalytic activity.

## Introduction

Polyurethanes are among the most widely used polymers in industry – in particular for specialty applications in furniture, construction, electronics, automotive and packaging. Typically, polyurethanes are obtained by polymerization of polyols with diisocyanates as co-monomer. However, the use of isocyanates is not considered sustainable^1^. Several methods are available for the synthesis of non-isocyanate polyurethanes (NIPU)^2,3^. A first step towards more sustainable polyurethanes was the synthesis of isocyanates using the Curtius rearrangement to avoid the use of phosgene, even though it has to be viewed critically, as it proceeds *via* toxic acyl azides^4^. Sustainable polyurethanes with backbones similar to the petroleum-based ones can be produced by transurethanization of carbamates^5–7^. Moreover, a sustainable synthesis of bio-based carbamates was performed by Lossen rearrangement of hydroxamic acids, which are activated *in situ* by dialkyl carbonates in the presence of catalytic amounts of tertiary amine bases (0.1–0.4 eq.)^8^. Still, NIPU synthesis from *bis*-cyclic carbonates (bCC) and diamines is arguably the most promising approach^9^, as the obtained molecular weights are typically higher than with the other approaches. The first route for the synthesis of five-membered carbonates is on the example of ethylene carbonate and involves phosgenation of ethylene glycol by phosgene or triphosgene, which is due to obvious reasons not suitable for a sustainable synthesis, as the original purpose of NIPUs is to avoid phosgene^10^. More sustainable approaches are typically based on the insertion of CO_2_ into epoxides, which are synthesized from renewable resources containing double bonds or from 1,2-diols.

For some carbonates, such as *e*.*g*. glycerol carbonate, the direct synthesis from the 1,2-diol and supercritical CO_2_ (_SC_CO_2_) has been established^11^, although the reaction is currently still not feasible on an industrial scale due to low conversions (32%)^2^. Another method utilizing supercritical CO_2_ was reported by M. Aresta *et al*. first obtaining the ketal of cyclohexanone and 1,2-ethanediol followed by transition-metal complex catalyzed carbonate formation in _SC_CO_2_ or with CO_2_ in organic solvents^12^. Other methods involving pressurized CO_2_ and 1,2-diols are performed in acetonitrile at 170 °C and catalyzed by metallic acetates^13^, by electrosynthesis^14^, or with diazabicycloundecen in dibromomethane^15^. The carbonate interchange reaction between 1,2-diols and ethylene carbonate or linear carbonates (dimethyl carbonate, diethyl carbonate or diphenyl carbonate)^16–18^ is more common and also used for the herein reported approach. A different type of transesterification can be achieved with urea and ZnO or various other solid catalysts releasing ammonia in the process^19^. Very interesting is the oxidative carbonylation with carbon monoxide using, for instance, palladium-based catalysts^20,21^, which is also the established synthesis of most linear carbonates. Alternatively to a two-step procedure (first synthesizing the epoxide and insertion of CO_2_ afterwards), olefins can be directly converted by oxidative carboxylation with a catalytic system of MoO_2_(acac)_2_, a quaternary ammonium salt and *tert*-butyl hydroperoxide as an oxidant in an one-pot multistep process^22^. Other methods available for this oxidative carboxylation were reviewed in 2011 by J. Sun and coworkers^23^. Methods involving other starting materials are of minor importance, *e*.*g*. from halohydrins^23^, substituted propargyl alcohols^24,25^, halogenated carbonates^26^, or linear oligo-carbonates^27–29^. Six- or seven- membered cyclic carbonates can be obtained from 1,3-diols or 1,4 diols by similar means as five-membered cyclic carbonates. While they provide a better reactivity due to a lower thermodynamic stability (six-membered cyclic carbonates react up to 60 times faster, seven-membered cyclic carbonates react up to 2,400 times faster)^9^, the synthesis of six- or seven- membered carbonates is significantly more difficult for exactly the same reason^30^. Still, K. Tomishige *et al*. reported a particularly efficient catalyst (CeO_2_) with high yields for six- membered carbonates (62–99%) from CO_2_ and diols in 2014^31^. However, a high excess (10 times) of 2-cyanopyridine was needed to remove water from the reaction mixture and obtain a high selectivity.

**Figure 1:**
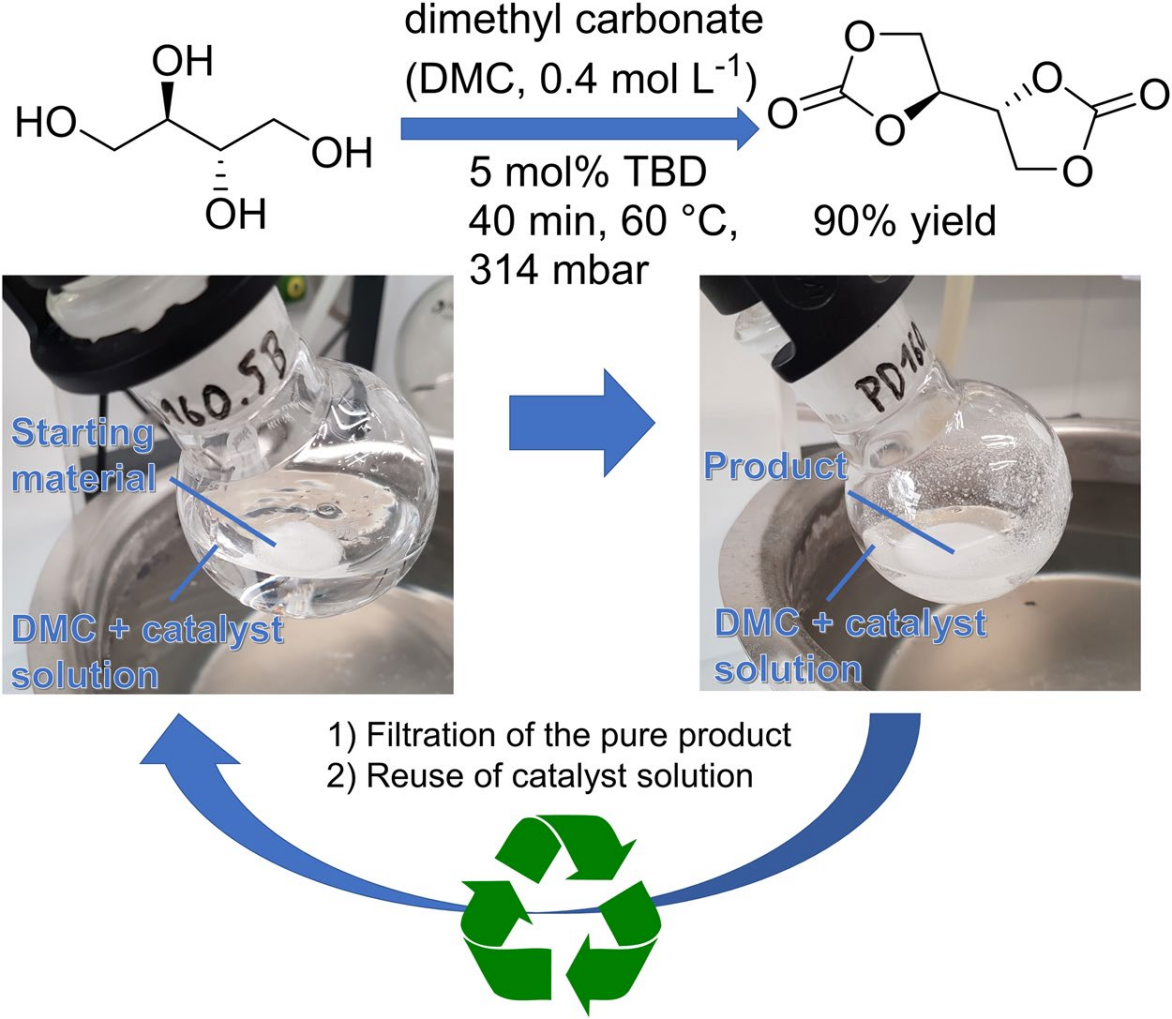
General reaction scheme of the reaction of erythritol to erythritol bis(carbonate). The pure product can be separated by filtration and the reaction mixture reused for another reaction cycle.

Erythritol *bis*(carbonate) (EBC) was already synthesized in 1960 from butadiene and patented by Union Carbide Whelan for polyurethane synthesis^32^, however it can also be synthesized in a more sustainable fashion by transesterification from the renewable sugar substitute erythritol. Erythritol is a narurally occuring sugar alcohol, which is present in many fruits and vegetables and used as a bulk sweetener. It is industrially produced by a fermentation process with the naturally occurring yeast *Moniliella pollinis* from aqueous solutions of glucose or sucrose^33^. Starting from erythritol, several routes to EBC are known. T. Griesser *et al*. achieved high yields up to 88%, however the phosgene based 1,1′-carbonyldiimidazole was used as carbonyl source^34^. Until now, the carboxylation of erythritol with dimethyl carbonate was claimed to be unfeasible due to low yields^35^, since in 2012 G. Rokicki *et al*. reported it as side reaction of an intramolecular etherification with only 10% yield^36^. They utilized an excess of dimethyl carbonate (DMC) at 70 °C and 226 mbar with K_2_CO_3_ as catalyst, only obtaining the desired EBC in 5% yield. In 2017, based on these results, R. Mülhaupt *et al*. used diphenyl carbonate (DPC) instead of DMC at 120 °C and 30 mbar in DMSO with Zn(OAc)_2_ as catalyst, thus avoiding the intramolecular etherification and obtaining EBC in 80–90% yield^35^. Additionally, the same method was later applied to sorbitol in order to obtain sorbitol tricarbonate^37^. Even though the yield is good, the synthesis lacks in sustainability regarding several aspects. DPC is far less sustainable than DMC. Aside from the classic unsustainable phosgenation of alcohols, both can be produced by oxidative carbonylation from the respective alcohol and carbon monoxide^38^. However, while phenol is typically produced in the cumene process from fossil benzene and propene^39^, methanol can be obtained *via* various different routes from renewables (*e*.*g*. from bio-derived synthesis gas or methane)^40^. Moreover, the process of the oxidative carbonylation of methanol is more efficient and involves cheaper catalysts (copper instead of palladium) and auxiliaries than the one of phenol. Industrially, the oxidative carbonylation of phenol is barely realized, while for methanol this route is established^38^. Here, we report on an efficient and sustainable synthesis of EBC based on DMC (Fig. 1).

## Results

To avoid the intramolecular ether formation described in literature^36^, a selective transesterification catalyst with high activity even at low temperatures was needed. TBD is known for such properties^41^ and was utilized in a moderate amount of 5 mol%. Erythritol was barely soluble in DMC, however at 60 °C, a small amount was soluble and thus successfully transesterified without observing any side reactions *via* GC-MS. By removing the methanol under reduced pressure, EBC precipitated from the reaction mixture and the overall equilibrium was shifted giving 90% yield after 40 minutes. After completion and cooling the mixture to room temperature, the product was simply filtered off and the mother liquor, which still contained DMC, the catalyst and dissolved trace amounts of product, could be reused in another reaction (compare Fig. 1). The recovery of the reaction mixture was performed eight times in triplicate with the same amount of reactant and solvent (i.e. adding fresh DMC and erythritol each time).

**Figure 2:**
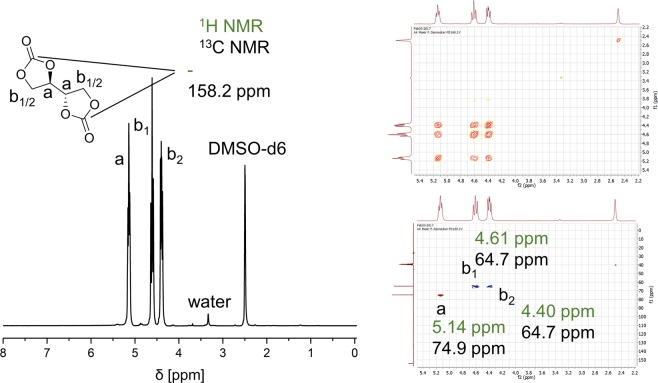
^1^H-NMR, HSQC and COSY of erythritol bis(carbonate). Chemical shifts of the ^1^H-NMR are depicted in green, correlated shifts of the ^13^C-NMR in black.

After the first run, the reaction time was shortened from 40 minutes to 25 minutes, which can be explained by product residue in the mixture promoting the crystallization. A similar decrease in reaction time could be obtained by adding 3.5 mol% product from the beginning, confirming that already present product promotes crystallization. After eight runs, an average yield of 90 ± 1% was observed, which fits to the initial 90%, indicating that the catalyst retained its activity. Even after storage of the reaction mixture overnight, the catalyst was still active and showed no signs of degradation. In contrast, evaporation of the mixture to dryness led to change in color to dark red after heating for additional 30 minutes, indicating the decomposition of the catalyst. Typically, after simple filtration and washing with DMC, the product was already highly pure, however, in case of remaining reactant or catalyst, the impurities might simply be removed by washing the product with water. In the ^1^H-NMR spectrum (see Fig. 2), three distinct peaks can be observed as the methylene group of signal b undergoes diastereotopic signal splitting due to hindered rotation, which can be confirmed by HSQC as signals b_1_ and b_2_ both couple with the same carbon at 64.7 ppm. The data of the ^1^H-NMR and ^13^C-NMR spectrum were in accordance with the literature^36^.

The simplicity of the synthesis and workup coupled with its sustainability clearly highlight the advantages compared to the literature procedure (see Table 1 and Scheme 1).

**Table 1:** Comparison of literature procedure and new procedure for the synthesis of erythritol bis(carbonate).

	Literature	New procedure
Solvent	DMSO	DMC
Carbonyl source	DPC	DMC
Reaction temperature	120 °C	60 °C
Reaction time	19 h	2 h
Pressure	30 mbar	314 mbar
Workup	recrystallization in acetone	direct filtration of the pure product
Catalyst	Zn(OAc)_2_*2H_2_O	TBD
Recycling	not possible	reaction mixture directly reusable without any purification.
Atom economy	31.6%	57.7%
Yield	80–90%	90%

**Figure 3:**
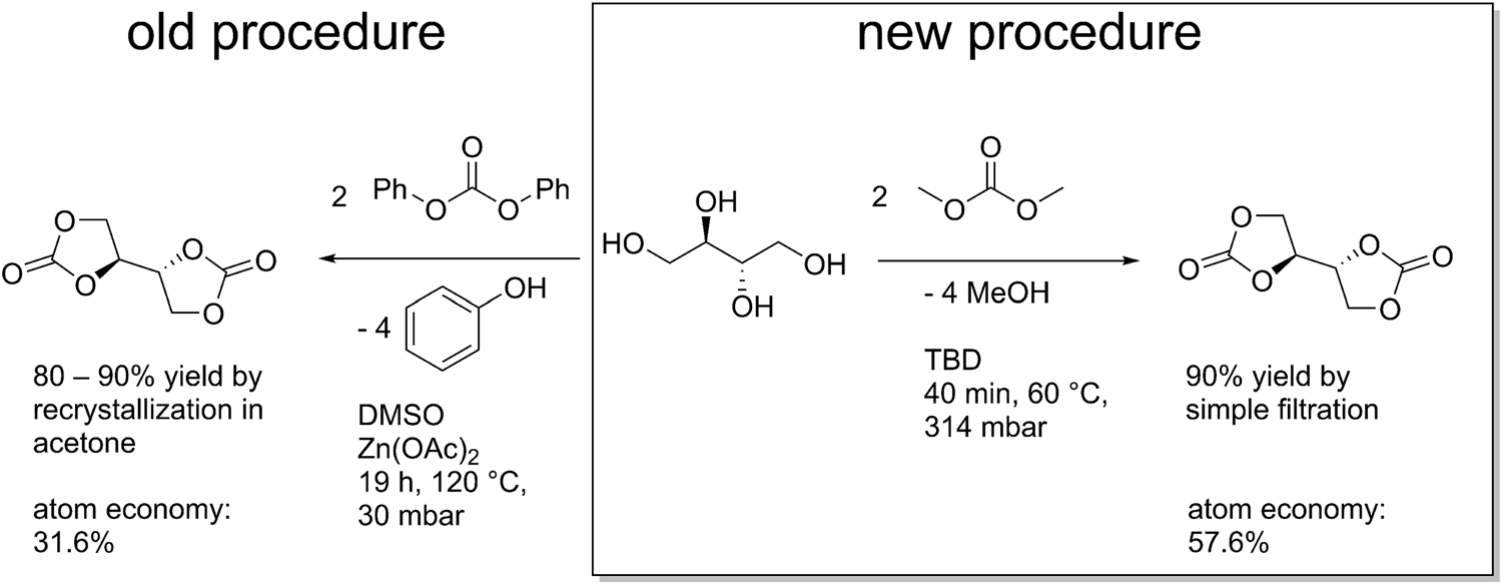
Schematic comparison of literature procedure and new procedure for the synthesis of erythritol bis(carbonate).

DMSO, the solvent for the literature procedure, achieves a total rating of “some issues” in GSK’s solvent sustainability guide, while DMC is among the few solvents with “few issues^42^”. DPC is, as mentioned above, a less sustainable carbonyl source compared to DMC due to its production but also because of the bad atom economy of the reaction itself (see Fig. 3). By using DMC, the atom economy could be improved from 31.6 to 57.7% compared to literature. The reaction temperature for the new procedure is far lower (60 °C compared to 120 °C) minimizing energy use. A shorter reaction time (2 h instead of 19 h) brings obvious advantages as well as a moderately (314 mbar) instead of a highly (30 mbar) reduced pressure. In fact, the reaction worked just as selective without any reduced pressure, however the product did not precipitate if a considerable amount of methanol formed during the reaction was present. The catalyst TBD is more expensive than Zn(OAc)_2_*2H_2_O (0.16 € g^−1^, *product number 383058-500G* compared to 6.80 € g^−1^, *product number 345571-5G*; Sigma Aldrich; checked 06.05.2018), but as it can be recovered and reused, the overall price might not be much higher. The workup consisted of a simple filtration compared to a recrystallization, which requires additional solvent and thus results in additional waste, as it cannot be reused or recycled. When testing the developed reactions conditions with sorbitol, another important renewable alditol that could yield a useful tricarbonate for NIPU synthesis, unfortunately, significantly lower conversion as well as selectivity was observed. Thus, the development of specific conditions for each alditol seems necessary. For instance, L. Avérous *et al*. reported a yield of up to 50% of (1R,4S,5R,6R)-6-(1,3-dioxolan-2-one-4-yl)-2,4,7trioxa-3-oxy-bicyclo[3.3.0]octane, a bCC from D-sorbitol obtained after intramolecular etherification, using 5 mol% TBD in DMC at 75 °C and a reaction time of 16 hours^43^.

In conclusion, erythritol *bis*(carbonate) – a valuable monomer for NIPU synthesis – was obtained from erythritol featuring mild reaction conditions and a facile workup in 90% yield. The reusability of catalyst and reaction mixture was demonstrated up to 8 times, while the catalyst showed no sign of decrease in activity. In principle, the developed procedure might enable a one-pot polymerization by directly adding the diamine to the reaction mixture due to its high selectivity as well as the presence of a TBD as a suitable catalyst, but keeping the catalyst in the monomer synthesis cycle even longer seems more practical and more sustainable.

## Materials and Methods

### Methods

GC-MS (electron impact, EI) measurements were performed on the following system: a Varian 431 GC instrument with a capillary column FactorFour VF-5 ms (30 m × 0.25 mm × 0.25 mm) and a Varian 210 ion trap mass detector. Scans were performed from 40 to 650 *m*/*z* at rate of 1.0 scans s^−1^. The oven temperature program was: initial temperature 95 °C, hold for 1 min, ramp at 15 °C min^−1^ to 220 °C, hold for 4 min, ramp at 15 °C min^−1^ to 300 °C, hold for 2 min. The injector transfer line temperature was set to 250 °C. Measurements were performed in the split – split mode (split ratio 50:1) using helium as carrier gas (flow rate 1.0 mL min^−1^).

NMR spectra (300 MHz for ^1^H- and at 75 MHz for ^13^C-measurements) were recorded on a Bruker AVANCE DPX spectrometer operating at 300 K. For all NMR-spectra the residual non-deuterated solvent (^1^H NMR) or CDCl_3_ (^13^C NMR) signal was used as internal standard. Infrared spectra (IR) were recorded on a Bruker Alpha-p instrument in a frequency range from 3998 to 374 cm^−1^ applying ATR technology.

ESI-MS spectra were recorded on a Q Exactive (Orbitrap) mass spectrometer (Thermo Fisher Scientific, San Jose, CA, USA) equipped with a HESI II probe to record high resolution electrospray ionization – MS (ESI-MS). Calibration was carried out in the m/z range of 74–1.822 using premixed calibration solutions (Thermo Fisher Scientific). A constant spray voltage of 4.7 kV and a dimensionless sheath gas of 5 were employed. The S-lens RF level was set to 62.0, while the capillary temperature was set to 250 °C.

### Synthesis of erythritol bis(carbonate)

In a 100 mL flask, erythritol (2.00 g, 16.4 mmol, 1.00 eq) and TBD (114 mg, 0.820 mmol, 0.05 eq.) were dispersed in 41 mL DMC and heated to 60 °C for 40 min at the rotary evaporator at 314 mbar. The crystalline erythritol dissolved completely after 30 min and after 35 min, a white precipitate was formed. The product was filtered off after the mixture was cooled down to room temperature and washed with dimethyl carbonate yielding a white powder (2.53 g, 90%).

^1^H-NMR (DMSO-d_6_, 300 MHz): δ (ppm) = 5.25–5.07 (m, 2 H, CH), 4.69–4.52 (m, 2H, CH_2_(a), diastereotopic signals), 4.47–4.31 (m, 2H, C*H*_2_, diastereotopic signals); ^13^C-NMR (DMSO-d_6_, 75 MHz): δ (ppm) = 158.2 (*C*O), 74.9 (*C*H), 64.7 (–*C*H_2_-); HRMS (ESI) of C_6_H_6_O_6_ [M + H]^+^ *m/z* calc. 175.0237, found 175.0234; IR (ATR): *ν* = 1803.7, 1779.0, 1545.5, 1476.1, 1381.2, 1299.6, 1206.5, 1144.6, 1067.0, 1030.9, 978.1, 896.2, 771.8, 739.1, 719.1, 532.1, 384.9 cm^−1^.

### Recovery experiments

In a 100 mL flask, erythritol (2.00 g, 16.4 mmol, 1.00 eq) and TBD (114 mg, 0.820 mmol, 0.05 eq.) were dispersed in 41 mL DMC and heated to 60 °C for 40 min at the rotary evaporator at 314 mbar. The crystalline erythritol dissolved completely after 30 min and after 35 min, a white precipitate was formed. The product was filtered off after the mixture was cooled down to room temperature and washed with 15 mL dimethyl carbonate, which was combined with the mother liquor, to obtain the product (2.53 g, 90%) and again 41 mL volume of a mixture containing the catalyst and non-precipitated product. Another batch of erythritol (2.00 g, 16.4 mmol, 1.00 eq) was dispersed in the mother liquor and the procedure was repeated for 7 additional times yielding on average 90 ± 1% (procedure carried out in triplicate) of the product.
